# Persistent and Widespread Pain Among African Americans Six Weeks after MVC: Emergency Department-based Cohort Study

**DOI:** 10.5811/westjem.2020.8.47450

**Published:** 2020-12-16

**Authors:** Francesca L. Beaudoin, Wanting Zhai, Roland C. Merchant, Melissa A. Clark, Michael C. Kurz, Phyllis Hendry, Robert A. Swor, David Peak, Claire Pearson, Robert Domeier, Christine Ortiz, Samuel A. McLean

**Affiliations:** * Warren Alpert Medical School of Brown University, Department of Emergency Medicine, Providence, Rhode Island; † Warren Alpert Medical School of Brown University, Department of Biostatistics, Providence, Rhode Island; ‡ Harvard Medical School, Department of Emergency Medicine, Boston, Massachusetts; § Warren Alpert Medical School of Brown University, Department of Obstetrics and Gynecology, Providence, Rhode Island; ¶ University of Alabama School of Medicine, Department of Emergency Medicine, Birmingham, Alabama; || University of Florida College of Medicine – Jacksonville, Department of Emergency Medicine, Jacksonville, Florida; # Oakland University William Beaumont School of Medicine, Department of Emergency Medicine, Royal Oak, Michigan; ** Harvard Medical School, Department of Emergency Medicine, Boston, Massachusetts; †† Wayne State University School of Medicine, Department of Emergency Medicine, Detroit, Michigan; ‡‡ St. Joseph Mercy Hospital, Department of Emergency Medicine, Ypsilanti, Michigan; §§ Rhode Island Hospital, Department of Emergency Medicine, Providence, Rhode Island; ¶¶ University of North Carolina – Chapel Hill, Department of Anesthesiology and Emergency Medicine, Chapel Hill, North Carolina

## Abstract

**Introduction:**

African Americans in the United States experience greater persistent pain than non-Hispanic Whites across a range of medical conditions, but to our knowledge no longitudinal studies have examined the risk factors or incidence of persistent pain among African Americans experiencing common traumatic stress exposures such as after a motor vehicle collision (MVC). We evaluated the incidence and predictors of moderate to severe axial musculoskeletal pain (MSAP) and widespread pain six weeks after a MVC in a large cohort of Black adults presenting to the emergency department (ED) for care.

**Methods:**

This prospective, multi-center, cohort study enrolled Black adults who presented to one of 13 EDs across the US within 24 hours of a MVC and were discharged home after their evaluation. Data were collected at the ED visit via patient interview and self-report surveys at six weeks after the ED visit via internet-based, self-report survey, or telephone interview. We assessed MSAP pain at ED visit and persistence at six weeks. Multivariable models examined factors associated with MSAP persistence at six weeks post-MVC.

**Results:**

Among 787 participants, less than 1% reported no pain in the ED after their MVC, while 79.8 (95% confidence interval [CI], 77.1 – 82.2) reported MSAP and 28.3 (95% CI, 25.5 – 31.3) had widespread pain. At six weeks, 67% (95% CI, 64, 70%) had MSAP and 31% (95% CI, 28, 34%) had widespread pain. ED characteristics predicting MSAP at six weeks post-MVC (area under the curve = 0.74; 95% CI, 0.72, 0.74) were older age, peritraumatic dissociation, moderate to severe pain in the ED, feeling uncertain about recovery, and symptoms of depression.

**Conclusion:**

These data indicate that African Americans presenting to the ED for evaluation after MVCs are at high risk for persistent and widespread musculoskeletal pain. Preventive interventions are needed to improve outcomes for this high-risk group.

## INTRODUCTION

Millions of Americans present to emergency departments (ED) each year for care after motor vehicle collisions (MVC).^1^ More than 90% do not have serious physical injury and are discharged home after their ED evaluation.^2^ Most individuals who have pain develop it in the “axial” regions (back, neck or shoulders).^3^ It is expected that most individuals will recover in the first several days after their accident, but a subset of individuals will develop persistent pain, which is pain that lasts beyond normal healing time.^4^–^6^ In addition, some post-MVC pain may become widespread over the body, which might portend a risk for the development of fibromyalgia, a condition associated with disability.^3^,^4^,^7^,^8^

Persistent or widespread musculoskeletal pain develops in at least 20% of non-Hispanic White individuals who experience “minor” MVCs.^5^,^6^ A recently published companion study of a cohort of non-Hispanic White, post-MVC ED patients, evaluated risk factors for persistent pain and demonstrated that initial severity of pain in the ED, neck pain, somatic symptoms (eg, nausea, dizziness), and pain catastrophizing were predictive of persistent pain at six weeks post-MVC.^4^ Although these findings from non-Hispanic White, post-MVC ED patients are important, they might not generalize to other races and ethnicities. There are reasons to believe the incidence of persistent or widespread pain among other racial and ethnic groups could be higher. In particular, African Americans are a historically understudied population that has consistently been shown to experience an increased burden of pain in other settings.^9^–^18^ Reasons for this increased burden of pain remain poorly understood. While some of this increased vulnerability to pain may be due to greater socioeconomic disadvantages,^19^ data from other clinical conditions suggests that worse health outcomes such as chronic pain among African Americans are not likely to be accounted for by socioeconomic differences alone.^20^

To our knowledge the incidence of persistent pain in African Americans experiencing an MVC has never been reported. In this study, we evaluated the incidence and predictors of persistent moderate or severe axial pain (MSAP) and widespread pain six weeks after MVC in a large cohort of African Americans who presented to the ED for initial care. We hypothesized that rates of MSAP and widespread pain would be higher than those previously observed in another prospective MVC cohort of non-Hispanic White Americans,^4^ and that pain, somatic symptoms, and acute psychological symptoms would be leading predictors of chronic pain outcomes.

## METHODS

### Study Design and Setting

This prospective, multicenter cohort study enrolled individuals (n = 930) who presented to the ED within 24 hours of an MVC and were discharged home after evaluation. Data were collected at the ED visit via patient interview and self-report surveys at six weeks after the ED visit via internet-based, self-report survey or telephone interview. Participants were enrolled at 13 EDs across the United States: University of Alabama at Birmingham Hospital (Birmingham, AL); University of Florida Health Jacksonville (Jacksonville, FL); Henry Ford Hospital (Detroit, MI); Sinai-Grace Hospital (Detroit, MI); Albert Einstein Medical Center (Philadelphia, PA); Detroit Receiving Hospital (Detroit, MI); St. Joseph Mercy Ann Arbor Hospital (Ypsilanti, MI), Medstar Washington Hospital Center (Washington DC); Boston Medical Center (Boston, MA); St. Joseph’s Regional Medical Center (Paterson, NJ), Spectrum Health Butterworth Hospital (Grand Rapids, MI); William Beaumont Hospital (Royal Oak, MI); and Baystate Medical Center (Springfield, MA) between September 2012–September 2016. The study was approved by the institutional review boards at each of the study sites, and all participants provided written informed consent. This study conforms with STROBE reporting guidelines and further details of study methodology are described elsewhere.^21^

**Population Health Research Capsule**

What do we already know about this issue?

*Persistent musculoskeletal pain develops in at least 20% of non-Hispanic Whites who experience minor injury after a motor vehicle collision (MVC)*.

What was the research question?

*What are the incidence and predictors of persistent and widespread pain 6 weeks after MVC among African Americans who presented to the emergency department (ED)?*

What was the major finding of the study?

*African Americans presenting to the ED for evaluation after MVC are at high risk for persistent and widespread musculoskeletal pain*.

How does this improve population health?

*Pain and psychological comorbidities could be important targets to prevent the transition from acute to chronic pain in this high-risk population*.

### Study Population

Patients 18–65 years old who presented to a study ED within 24 hours of an MVC and were unlikely to require hospitalization were screened for study eligibility. We excluded patients who were admitted to the hospital, had any fractures other than phalangeal fractures, had more than four lacerations requiring sutures or a single laceration more than 20 centimeters in length, or had intracranial or spinal injuries. Spinal injury was defined by the presence of a fracture, dislocation, or new neurologic deficit. We selected these criteria to have a study sample with relative homogeneous injuries (eg, isolated musculoskeletal pain only) and to match enrollment criteria in a companion cohort of non-Hispanic White MVC patients.^4^ Patients who were not alert and oriented also were excluded, but those with loss of consciousness and return to normal were eligible for the study. We also excluded pregnant patients, inmates, and patients unable to read and understand English. Enrollment was limited to individuals who self-identified as non-Hispanic Black or African-American, in order to focus on a traditionally understudied, high-risk population. This study was also one of two cohort studies evaluating pain pathogenesis after MVC. The first cohort was conducted in non-Hispanic White patients of self-identified European-American ancestry and the second cohort (presented in this investigation) recruited non-Hispanic Black patients of self-identified African ancestry. The rationale for separate cohorts was because the aims of the parent study included genetic analyses, and studies involving genetic analyses require a more homogeneous population.^22^ The non-Hispanic White cohort closed first and results have been published elsewhere^4^; the analyses are therefore not included here to avoid duplicated publication.

### Baseline Measures

During the ED interview, participants completed a structured research assistant (RA)-administered survey using web-based forms on laptop computers. This interview elicited information about pre-MVC characteristics and also contained a series of questions related to the patient’s current and past pain and other pain-related characteristics. The initial interview captured sociodemographic characteristics (eg, age, gender, education level, income level, employment status, marital status), pre-MVC health status, medical history, medication history, and MVC characteristics (eg, position in the vehicle, speed, seatbelt use). Pain assessments included locations of pain, number of body regions with pain, severity of pain in each body region, and whether or not the pain was related to the MVC. Severity of pain was assessed using the “0–10” numerical rating scale (NRS), a valid assessment of pain in ED patients.^23^ Additional pain-related symptoms were assessed using the following instruments: post-MVC psychological distress (per the Peritraumatic Distress Inventory^24^); dissociation (distorted memory, awareness, or perception of trauma characterized per Michigan Critical Events Perception Scale^25^); anger (per State-Trait Personality Inventory Form Y^26^); and somatic symptoms (per Pennebaker Inventory for Limbic Languidness^27^). Each of these assessments has been detailed elsewhere along with complete study methodology.^21^ W abstracted data regarding participants’ injuries from the ED health record. Baseline measures from these instruments were used as possible candidate predictors for the modeling of the outcomes of MSAP and widespread pain at six weeks post-MVC.

### Outcome Definitions: Moderate to Severe Axial Pain, Widespread Pain

We selected the six-week follow-up time point for evaluation because chronic pain trajectories after MVC are generally established within 6–8 weeks of MVC,^19^,^28^,^29^ and because this facilitated comparison with six-week outcomes of non-Hispanic European Americans reported in a previous study employing similar design and methods.^4^ Pain location and severity at six-week follow-up were assessed using the same methods employed at baseline evaluation, except that if a participant reported pain in a body region they were also then asked whether the pain was due to the MVC. As in our previous studies, we used only pain reported as due to MVC in outcome analyses. MSAP was defined by a pain score of ≥ 4 in the neck, shoulder, or upper or lower back regions^30^; and widespread pain was defined by the presence of axial pain plus pain in one or more body regions above and below the waist and on the left and right side.^31^

### Analysis Plan

We performed descriptive and inferential statistics and predictive modeling using Stata MP 13.0 statistical software (StataCorp, 2013, College Station, TX) and SAS 9.1.3 software (SAS Institute Inc., 2016, Cary, NC). Chi-squared tests were performed to evaluate for possible selection bias due to loss to follow-up by comparing the sociodemographic characteristics of those participants retained in the cohort at six weeks vs those who did not complete their six-week follow-up.

### Predictive Modeling

We developed a list of candidate predictors based on substantive knowledge of factors likely to be associated with the development of persistent or widespread pain following a MVC.^3^,^4^,^7^,^8^,^29^,^32^–^35^ These candidate predictors were sociodemographic characteristics, MVC characteristics, pain and somatic symptoms reported immediately after the MVC, and baseline psychological and cognitive characteristics. Log-binomial regression with a robust error estimation method was used to evaluate the association (risk ratio) between each candidate predictor and the primary outcomes of MSAP and widespread pain at week six post-MVC. If a candidate predictor was a scale from a survey instrument, we categorized the scores according to established cut-offs or divided them into tertiles based on the score. Forest plots were used to depict the estimates of the risk ratio and associated 95% confidence intervals (CI) for the primary outcomes and the candidate variables.

Predictors showing evidence of association with the outcomes in bivariate analyses were used as candidate predictors in multivariate analyses (p<0.20). We performed multivariate analyses using a least absolute shrinkage and selection operator (LASSO). LASSO (log-binomial) regression was chosen for the following reasons: 1) we aimed to replicate the analyses performed in the companion cohort of non-Hispanic White MVC patients in order to draw qualitative comparisons between the two groups and LASSO was used in that analysis; 2) LASSO performs automatic variable selection; and 3) LASSO can result in selection of fewer variables than other techniques leading to a model that is easier to interpret. We used a 10-fold cross-validation approach to limit model over-fitting and selected the model with the lowest root-mean squared error as the final model.^36^ The maximum number of predictors allowed in our predictive model was based on the “rule of 10s” (10 events per predictor).^37^

We tested model performance using the area under the receiver operating characteristic curve (ROC) and calibration plots. An area under the ROC curve (AUC) value of 0.70 or greater was considered to have a fair predictive ability.^38^ Because different modeling techniques could yield a different set of predictors or perform differently in a given dataset, we conducted a sensitivity analysis using two other common modeling approaches: forward stepwise regression; and recursive partitioning (ie, classification and regression trees [CART]). Performance in these models was assessed using the AUC and compared to the results from the principal analyses.

### Missing Data

Predictors of the outcome were recorded with minimal missingness; over 95% of the baseline covariates had no more than 1% missing values, and the maximum proportion of missing for any baseline covariate was 6.4%. Missingness in the outcome variables also was minimal. We did not identify any systematic reasons for the missing values and therefore believe the data are missing completely at random; hence, the main analyses used only the complete cases. We performed a sensitivity analysis to evaluate the impact of missing data on our point estimates by repeating the primary analyses in a multiply imputed dataset. We performed multiple imputation of missing covariate data using chained equations and by specifying the conditional models for all of the variables with missing values.^39^,^4^

## RESULTS

### Participant Characteristics

**Figure 1:**
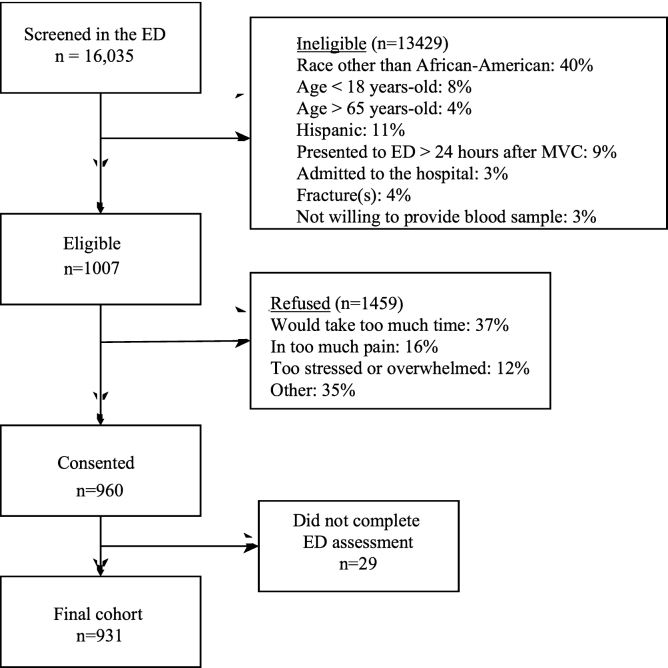
Screening, eligibility and enrollment of individuals. *ED*, emergency department; *MVC*, motor vehicle collision.

Figure 1 displays the screening, eligibility, and enrollment of the study cohort. Of the 931 individuals enrolled, six-week follow-up data were obtained in 84.6% of participants (n = 787); the remainder were lost to follow-up. The majority of participants were women (> 60%), the median age was 32 years (interquartile range [IQR] 24, 45), 99% of the participants had isolated musculoskeletal injuries (< 1% also had phalangeal fractures), and most participants had an annual household income of less than $40,000 per year. We obtained six-week follow-up data in 787/931 (85%) of participants; there were no significant differences in the sociodemographic characteristics of participants who did and did not follow-up. Table 1 provides an overview of participant characteristics.

### Participant Pain Characteristics in the ED

Table 2 shows pain-related characteristics reported by participants during the ED visit and at the six-week follow-up. At baseline, less than 1% of participants reported no pain in the ED and nearly 95% reported pain scores ≥ 4; the median pain score was 7.5 (IQR 6, 9). Approximately 80% (95% CI, 77.1, 82.2%) of all participants reported moderate to severe pain in the back, neck, or shoulders (axial). The median number of body regions with pain was 5 (IQR 2, 8), and 28.3% (95% CI, 25.5, 31.3%) had widespread pain in the ED. Reported pain (pain scores and proportion of patients with moderate or severe pain) was decreased at the six-week follow-up, but participants reported more body areas with pain and more somatic symptoms.

### Participant Pain Characteristics at Six-week Post-MVC Follow-up

**Figure 2:**
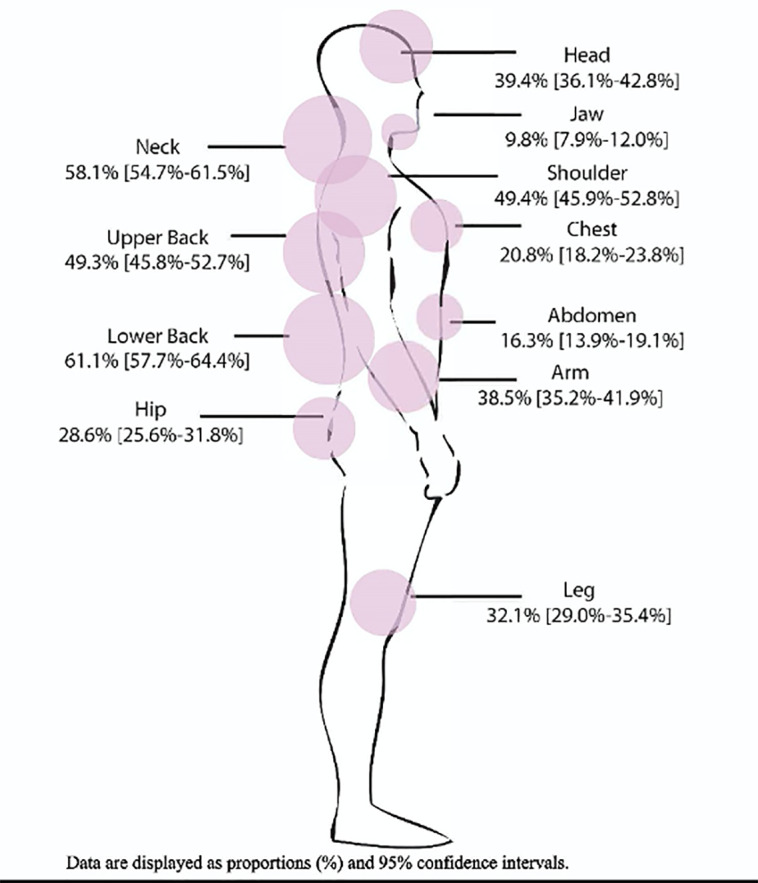
Patients reporting moderate to severe pain by body region.

Among the 787 participants completing the six-week follow-up, 78% reported moderate to severe pain in at least one body area. Figure 2 depicts the proportion of participants reporting moderate to severe pain by body region; 67% (95% CI, 63.9, 69.9%) reported moderate to severe pain in the axial region. Widespread pain was reported by 30.5% (95% CI, 27.7, 33.6%) of participants.

### Associations Between Demographic and Collision Characteristics and Adverse Pain Outcomes at Six-weeks Post-MVC Follow-up

**Figure 3:**
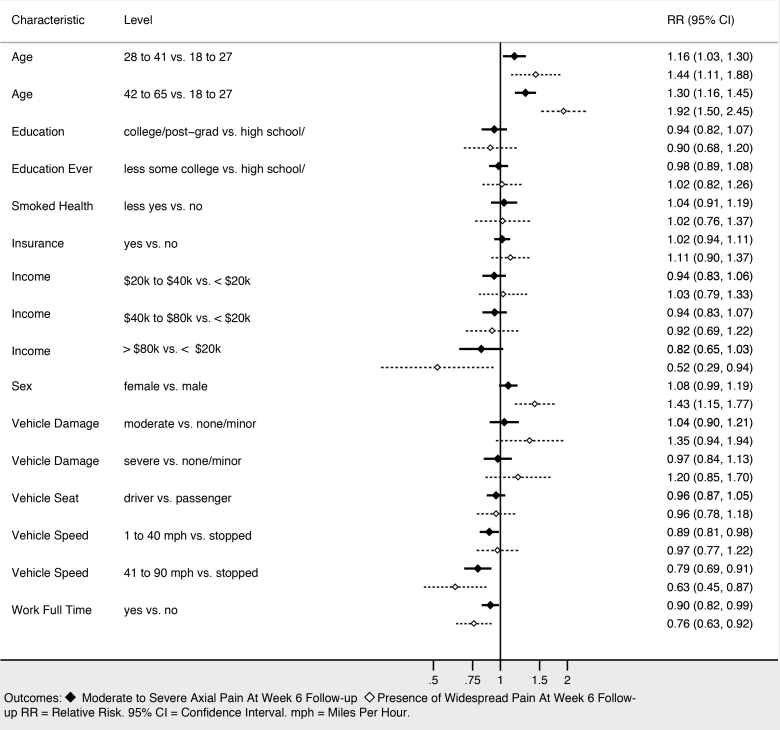
Sociodemographic and crash characteristics associated with persistent pain at six weeks post-motor vehicle collision (univariable).

**Figure 4:**
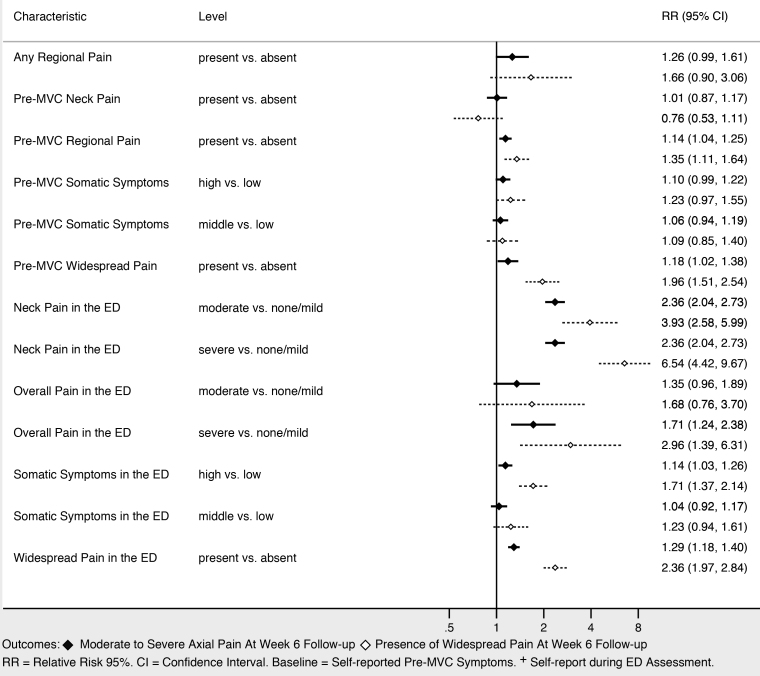
Pain and somatic symptoms associated with persistent pain at six weeks post-motor vehicle collision.

**Figure 5:**
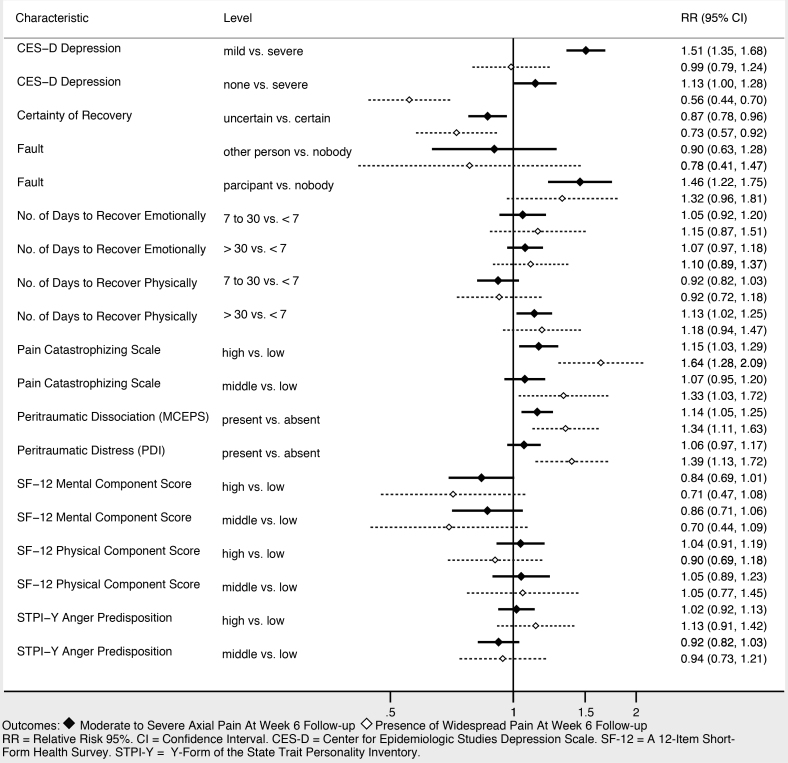
Psychological and cognitive characteristics associated with persistant pain at six weeks post-motor vehicle collision.

Sociodemographic characteristics associated with moderate to severe axial musculoskeletal pain and widespread pain at six weeks included increasing age, female gender, and not working full time (Figure 3). Higher income was associated with being protective against widespread pain, and showed a trend toward being protective against moderate to severe axial musculoskeletal pain. Collision characteristics were generally not associated with adverse pain outcomes, with the exception that being in a vehicle traveling 41–90 miles per hour vs a stopped vehicle was associated with increased risk of both moderate to severe axial musculoskeletal pain and widespread pain. Severity of pain in the ED (Figure 4), the presence of catastrophizing symptoms (a negative, exaggerated response to actual or anticipated pain) and peritraumatic distress (Figure 5) were all associated with increased risk of both moderate to severe axial musculoskeletal pain and widespread pain. Contrary to our a priori belief, milder (vs severe) depressive symptoms and being certain of recovery were associated with an increased risk of MSAP in these bivariate analyses, but this did not hold true in the multivariable predictive model.

### Multivariable Predictive Models of Persistent Pain at Six Weeks Post-Motor Vehicle Collision Follow-up

In multivariable analyses, increasing age, history of significant depressive symptoms (yes/no), presence of peritraumatic dissociation (yes/no), moderate to severe pain in the ED (NRS ≥ 4), and being uncertain of recovery in the ED most efficiently predicted continued MSAP six weeks after MVC (AUC = 0.74; 95% CI, 0.72, 0.76). In multivariable analyses, widespread musculoskeletal pain six weeks after MVC was most efficiently predicted by increasing age, female gender, vehicle speed, history of pre-MVC neck pain, overall pain severity in the ED, widespread pain in the ED, presence of somatic symptoms, depressive symptom severity in the ED, and pain catastrophizing (AUC = 0.74; 95% CI, 0.72, 0.76).

In a sensitivity analysis that re-examined the main analyses using two alternative modeling approaches (recursive partitioning, CART, and forward stepwise regression) model performance was similar for each approach and for both outcomes (AUCs ranged from 0.71 to 0.76). In addition, all six models that we constructed in the primary and sensitivity analyses (3 approaches × 2 outcomes) displayed adequate calibration (generally linear line along the 45° axis (R^2^ > 0.9)), indicating good agreement between the observed outcomes and predictions. For moderate to severe axial musculoskeletal pain, the three modeling approaches identified all of the predictors included in the LASSO model (increasing age, history of significant depressive symptoms (yes/no), presence of peritraumatic dissociation (yes/no), moderate to severe pain in the ED (NRS ≥ 4), and being uncertain of recovery in the ED). In addition, recursive partitioning also identified vehicle speed and number of somatic symptoms as predictors and stepwise regression for moderate to severe axial musculoskeletal pain also identified education as a predictor. All three approaches yielded the same minimum set of predictors for the development of widespread pain: increasing age, female gender, vehicle speed, history of pre-MVC neck pain, overall pain severity in the ED, widespread pain in the ED, presence of somatic symptoms, depressive symptom severity, and pain catastrophizing.

## DISCUSSION

Multiple lines of evidence would suggest that African Americans experience a greater burden of adverse outcomes than non-Hispanic White Americans (a much more commonly studied group), and yet this study constitutes the first large-scale, prospective study of pain outcomes among African Americans experiencing an MVC. African Americans continue to be underrepresented in many fields of clinical research. The greatest utility of this study is that it identifies the profound burden of acute and persistent musculoskeletal pain experienced by African Americans presenting to the ED after MVC. Nearly 95% of African Americans presenting to the ED after MVC, at 13 ED sites across the US, had acute moderate or severe musculoskeletal pain. Even more striking, nearly 80% of African Americans experienced persistent moderate or severe MVC-related pain and more than 3 in 10 individuals experienced MVC-related widespread pain at the six-week follow-up. These rates are over twice that previously reported in our large companion cohort of non-Hispanic White MVC patients that used similar methods,^4^ and suggest an urgent need for further studies to understand chronic pain pathogenesis and improve outcomes in this high-risk group.

In other settings, African Americans have been found to have relatively increased sensitivity to experimentally induced pain as compared to non-Hispanic Whites.^9^–^18^ Reasons for this increased burden of pain remain poorly understood. While some of this increased vulnerability to pain may be due to greater socioeconomic disadvantages, data from other clinical conditions suggests that worse health outcomes such as chronic pain among African Americans are not likely to be accounted for by socioeconomic differences alone.^41^

Increasing age, female gender, and the presence of pain catastrophizing and peri-traumatic distress were associated with persistent pain, unadjusted for other characteristics. More severe depressive symptoms reported in the ED were paradoxically associated with improved outcomes. The converse was true in the multivariable model, suggesting the presence of confounding in the unadjusted analyses. In multivariable predictive modeling, acute pain, increasing age, and a history of depression were predictive of both moderate to severe musculoskeletal pain and widespread pain six weeks after MVC in this Black cohort. This information may also be informative for subsequent interventions. For example, non-opioid medications such as serotonin-norepinephrine inhibitors have shown promise in the prevention and treatment of chronic pain, and their impact appears to be modified by the presence of depression: they are more effective in depressed patients.^42^

While a majority of patients in this study had ongoing MSAP at week 6, a smaller but still sizeable proportion of participants had widespread pain. Predictors of widespread pain were different than persistent moderate to severe pain in this cohort, suggesting that the pathogenesis of widespread pain is different. Gender, a history of chronic pain, having widespread pain, and somatic symptoms while in the ED were predictive of widespread pain at six weeks, but not MSAP. These predictors align with factors that are known to be associated with fibromyalgia, a condition associated with disability and impaired function.^43^ In addition, different prevalence and predictors may indicate that different interventions are needed in the subgroup at risk for widespread pain compared to those who are not at risk for widespread pain development.

There were some similarities in predictors of pain in both the Black MVC patients in this study and the previously published non-Hispanic White cohort.^4^ Specifically, the severity of acute pain in the ED was the only variable to appear in all models for both cohorts. Since acute pain appears to be a ubiquitous predictor in the development of persistent moderate to severe or widespread pain, it is important to further investigate what impacts the development of acute pain and whether interventions that improved acute pain also improve more distal pain outcomes. In a secondary analysis of these two MVC cohorts, African Americans were identified to have a higher burden of acute pain in the ED.^44^ The differences in acute pain may be directly related to the development of persistent MSAP and widespread pain weeks later. It is unclear whether these differences persist over time (months or years later) and whether interventions that improve acute pain in the ED are capable of altering the transition to chronic pain. An ongoing large and diverse cohort of trauma patients (n = 5000) recruited across the US is currently underway (the AURORA study) and may be able to answer some of these important remaining questions.^45^

## LIMITATIONS

This investigation has several limitations. Although conducting the study at multiple EDs likely increases its external validity, the findings might not be similar among those who declined study participation or whose sociodemographic characteristics were different than those who participated in the study (eg, low health literacy). It is unknown, however, whether the study findings are externally valid to other clinical settings (eg, primary care) and other racial or ethnic groups. In addition, the study relies on self-report and multiple questionnaires; self-reported outcomes are subject to reporting bias. Pain is subjective as are many of the other measurements, so no objective measures are possible for much of the data collected. However, the instruments used are commonly employed in several other studies, which permit comparisons to other settings. We performed multiple unadjusted bivariate associations. This was meant to be exploratory in nature and provide formative data for other work; multiple associations should be interpreted cautiously.

In addition, understanding factors that predict the outcome does not imply a causal relationship and should be interpreted cautiously. In addition, because the outcomes were common, a predictive model has limited utility as a risk-stratification tool in clinical practice. Rather, the predictive model provides insight into the risk of pain development and might provide substantive information for future interventions aimed at reducing the transition to chronic pain. Model performance and metrics of performance were not improved with other modeling techniques. This observation suggests that additional improvements in model performance might require other unmeasured predictors (eg, genetics) or using other techniques such as machine-based algorithms with the ability to “learn” complex interactions between predictors.

## CONCLUSION

In a large study of Black ED patients receiving care after motor vehicle collisions, moderate to severe axial musculoskeletal pain was the norm and widespread pain was also common six weeks later. The incidence rates of both types of pain were higher than has been reported in a cohort of non-Hispanic White patients with a similar trauma exposure. This finding suggests that African Americans are at higher risk than non-Hispanic White patients regarding the transition to chronic pain and that further research in this population is needed. Some factors, such as age, vehicle speed, and history of chronic pain help to predict risk, but are clearly not targets for intervention. Conversely, pain and certain psychological characteristics (eg, depression) could be important in future interventions aimed at targeting the transition from acute to chronic pain.

## Figures and Tables

**Table 1:** Characteristics of participants at enrollment and retained at six-week post-motor vehicle collision follow-up.

	Baseline (n =931)		6-week follow-up (n=787)		P-value
			
	n	%	n	%	p<
Age					0.93
18–27	346	37.2	287	36.5	
28–41	292	31.4	253	32.2	
42–65	292	31.4	247	31.4	
Gender					0.33
Male	352	37.9	280	35.6	
Female	578	62.2	507	64.4	
Education					0.80
High school or less	370	40.1	305	39.0	
Some college or trade school	380	41.2	321	41.1	
College/postgraduate degree	173	18.7	156	20.0	
Annual income					0.99
<$20,000	253	34.7	216	34.6	
$20,000 to $40,000	222	30.5	189	30.2	
$40,000 to $80,000	194	26.6	168	26.9	
>$80,000	60	8.2	52	8.3	
Works full time					
No	426	46.0	358	45.6	
Yes	501	54.1	428	54.5	

**Table 2:** Motor vehicle collision-related (MVC) characteristics at baseline in the emergency department and six-week post-MVC follow-up.

Characteristics	ED (Baseline) n=787	6 Weeks n=787
Pain intensity (NRS)	7.2 (7.1 – 7.4)	6.0 (5.8 – 6.2)
Body regions with pain (n)	5.9 (5.6 – 6.2)	7.7 (7.3 – 8.1)
Overall pain (%)		
No	0.7 (0.3 – 1.5)	8.4 (6.6 – 10.5)
Mild	4.7 (3.5 – 6.3)	13.1 (10.9 – 15.7)
Moderate	27.2 (24.4 – 30.2)	28.1 (25.1 – 31.4)
Severe	67.4 (64.3 – 70.4)	50.4 (46.9 – 53.9)
Moderate to severe axial pain (%)		
Yes	79.8 (77.1 – 82.2)	67.0 (63.9 – 69.9)
Widespread pain (%)	28.3 (25.5 – 31.3)	30.5 (27.7 – 33.6)
Somatic symptoms (n)	3.5 (3.5 – 3.7)	9.1 (8.6 – 9.6)

Continuous data are presented as means and 95% confidence intervals (CI); categorical data are displayed as proportions (%) and 95% CIs.

*ED*, emergency department; *NRS*, numerical rating scale.
